# Homocysteine-Enhanced Proteolytic and Fibrinolytic Processes in Thin Intraluminal Thrombus and Adjacent Wall of Abdominal Aortic Aneurysm: Study In Vitro

**DOI:** 10.1155/2018/3205324

**Published:** 2018-12-12

**Authors:** Aldona Siennicka, Marta Zuchowski, Kornel Chełstowski, Miłosław Cnotliwy, Jeremy Simon Clark, Maria Jastrzębska

**Affiliations:** ^1^Department of Laboratory Diagnostics, Pomeranian Medical University, Szczecin, Poland; ^2^Department of Vascular Surgery and Angiology, Pomeranian Medical University, Szczecin, Poland; ^3^Department of Clinical and Molecular Biochemistry, Pomeranian Medical University, Szczecin, Poland

## Abstract

Homocysteine (Hcy) may affect the pathogenesis of abdominal aortic aneurysms (AAAs) through enhancement of proteolysis and an impaired coagulation/fibrinolysis system. Intensified haemostatic capacity may promote local proteolytic degradation of the aortic wall. This study aimed to examine the effects of Hcy on haemostatic and proteolytic processes in samples of thick and thin fragments of the ILT and underlying walls.* Subjects and Methods*. Thirty-six patients who underwent AAA surgery were enrolled. Aneurysm tissue sections were incubated with DL-Hcy (100 and 500 *μ*mol/L) in a series of experiments and analyzed for concentration/activity of proteolytic and haemostatic markers by enzyme-linked immunosorbent assay.* Results*. Incubation of wall underlying thin ILT segments (B) with DL-Hcy resulted in an increase of active MMP-2 levels compared to control tissue (9.54 ± 5.88 versus 7.44 ± 4.48, p=0.011). DL-Hcy also induced t-PA and plasminogen concentration increases in thin thrombus sections (B1) compared to control tissue (respectively: 1.39 ± 1.65 versus 0.84 ± 0.74, p=0.024; 11.64 ± 5.05 versus 10.34 ± 5.52, p=0.018). In contrast, wall adjacent to thick thrombus segments (A) showed decreases in MMP-2 and TF activities compared to control (respectively, 5.89 ± 3.39 versus 7.26 ± 5.49, p=0.046; 67.13 ± 72.59 versus 114.46 ± 106.29, p=0.007). In thick ILT sections (A1), DL-Hcy decreased MMP-2 activity and t-PA and plasminogen concentrations compared to control tissue (respectively, 2.53 ± 2.02 versus 3.28 ± 2.65, p=0.006; 0.67 ± 0.57 versus 0.96 ± 0.91, p=0.021; 9.25 ± 4.59 versus 12.63 ± 9.56, p=0.017). In addition, analysis revealed positive correlations at all sites between activities/concentrations of MMP-2, TF, and PAI-1 measured in control tissues and after incubation with DL-Hcy.* Conclusions*. These data indicate the potential for excess Hcy to enhance damage of arterial wall in thinner AAA segments as a result of the increased activity of MMP-2 and fibrinolytic factors.

## 1. Introduction

Abdominal aortic aneurysm (AAA) is an abnormal, irreversible dilatation of the abdominal aorta, with ensuing mortality associated with aneurysm rupture with approximately 70-80% incidence. The pathogenesis of AAA appears to be multifactorial [1, 2]. An altered coagulation/fibrinolysis system and proteolytic activity may be some of the factors promoting the development of AAA and result in segmental weakening of the vascular wall and further dilatation, which eventually may lead to AAA rupture [3, 4].

A number of studies have demonstrated that elevated total plasma levels of homocysteine (Hcy), a sulphur amino acid derived from methionine, is a strong independent risk factor of cardiovascular diseases. Moreover, hyperhomocysteinemia (HHcy) has been associated with the size, diameter, and expansion rate of AAA [5–9]. Previous studies have shown that the levels of plasma Hcy were significantly increased in AAA patients compared to control individuals and correlated significantly with the disruption of the internal elastic lamina [5, 10–12]. HHcy can mediate the formation of AAA through several different mechanisms, e.g., via endothelial dysfunction, enhancement of proteolysis, impaired fibrinolysis, and oxidative damage, all of which are crucial steps in AAA pathogenesis. It is documented that Hcy may activate the zymogen pro-MMP-2 to the active form metalloproteinase-2 (MMP-2). Moreover, HHcy has been shown to not only activate MMPs but also alter the expression of tissue inhibitors of metalloproteinase (TIMPs), which results in abnormal MMP activity which may be relevant to AAA [13, 14]. Some studies suggest that HHcy leads also to alterations in coagulation and fibrinolysis. Homocysteine has been reported to exhibit antifibrinolytic properties, by reduction of tissue-type plasminogen activator (t-PA) binding to endothelial cells, but independent of t-PA expression, and promote thrombosis by inducing tissue factor (TF) and stimulating plasminogen activator inhibitor 1 (PAI-1) gene expression and secretion in the vasculature [15–18]. Taken together, most of these observations suggest that HHcy modifies endothelial function in a prothrombotic manner and favours thrombogenesis. However, the mechanism underlying the deterioration of vascular injury by elevated levels of Hcy remains unclear.

Intraluminal thrombus (ILTs) are found in the majority of AAAs. The size of an ILT varies, as does the extent to which it fills the lumina of the AAA [19–21]. The ILT can either be eccentrically located or cover the entire internal wall of the AAA. ILT thickness has previously been indicated as an important local factor correlated with effects on the underlying wall and adsorption of plasma components. An ILT also functions as a site of protease release and activation, with subsequent degradation of the extracellular matrix [19, 21, 22]. At present, the role of ILT in the progression and potential rupture of AAA is still controversial. Some investigators think that thick ILT could accelerate AAA rupture [23, 24]. In contrast, a lot of evidence suggests that the biologically active segment of an ILT seems to be limited to the luminal layer in direct contact with the aneurysm wall and that proteolytic processes occur more intensively within the AAA segment closer to the luminal layer [25–27]. In our previous work we noticed that the part of an aneurysm associated with thin segments of ILT was characterized by a higher concentration of MMPs and a higher activity of coagulative processes in comparison to parts associated with thick thrombus segments [27, 28]. Moreover, it was reported that in ruptured aneurysms the ILT was significantly thinner compared to nonruptured [19]. Together, these results support the hypothesis that the thickness of the ILT affects the chances of rupture of an AAA, and the activity of proteolytic and fibrinolytic processes show significant differences between thin and thick ILT segments.

Based on earlier reports, we hypothesized that Hcy may be one of the factors that intensifies proteolytic and haemostatic systems in segments of AAA in different ways when adjacent to thick and thin segments of ILT and that ILT thickness, together with Hcy activity, contributes to the chances of AAA rupture. This study aimed to (i) investigate the in vitro effect of elevated levels of DL-Hcy on proteolytic and haemostatic activity in thick and thin thrombus segments and aortic walls directly underlying thrombus, (ii) analyze correlations between proteolytic and haemostatic parameters after incubation with or without Hcy within individual aneurysm compartments. This study could contribute to further improvements in aneurysm monitoring and progress in predictive tools other than simply the size of an AAA.

## 2. Methods

### 2.1. Patients

Aneurysmal aortic specimens were surgically obtained from thirty-six patients (mean age 71 ± 8, age range 55 to 81 years) treated electively for AAA repair. There were 27 (75%) men and 9 (25%) women. The most important inclusion criteria were the presence of thick (≥25 mm) as well as thin (≤10 mm) intraluminal thrombus in one sac of aneurysm. Exclusion criteria included liver dysfunction, haematological disorders, known history of renal dysfunction, and chronic anticoagulation therapy. All patients signed informed consent. This study was approved by the Ethical Committee of the Pomeranian Medical University and conformed to the Helsinki declaration.

### 2.2. Measurement of AAA Diameter

The diameter of the AAA and ILT thickness was measured by computed tomography (CT) scan. An aorta with an infrarenal aortic diameter greater than 30 mm was defined as an abdominal aortic aneurysm. CT scans were used to measure aortic lumina at the level of maximal dilatation. The difference between maximal aortic lumen diameter and diameter of the lumen at the ILT was used to calculate ILT thickness.

### 2.3. Total Homocysteine Levels in Serum

Fasting blood samples were collected, prior to surgery, from patients with AAA. Measurements of concentrations of tHcy (to correlate with AAA diameter, ILT thickness, and haemostatic parameters) were performed with serum prepared from whole blood by centrifugation for 30 min at 3000 x g at 4°C, measured using Chemiluminescent Microparticle Immunoassay (CMIA); ARCHITECT System Homocysteine, Abbott Diagnostics, Lake Bluff, Illinois, USA.

### 2.4. Tissue Sampling

**Figure 1:**
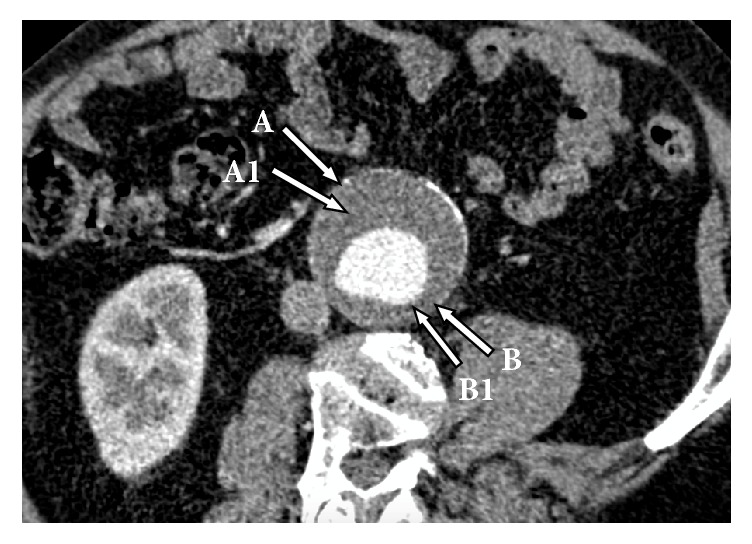
Typical computed tomography image of patient suffering from an abdominal aortic aneurysm, demonstrating the sites of sampling: A, wall adjacent to thick thrombus segment; A1, thick thrombus section; B, wall underlying thin thrombus section; B1, thin thrombus section.

In order to assess the effects of DL-homocysteine, as a potential stimulator for selected parameters in vitro, tissue samples were taken from the study group at maximal diameters of visually thick thrombus and thin thrombus segments of AAA at the time of surgery. Before excision of specimens, a laser micrometer was used to measure the thrombus thickness at each site. Tissue samples were taken from the middle 1/3 of the longitudinal dimension of the AAA sac (A and B sites) at the same level as for the thrombus sections (A1 and B1) (Figure 1). The aneurysm samples therefore comprised four groups: thick thrombus section (A1); thin thrombus section (B1); wall (A) adjacent to thick thrombus segment; wall (B) adjacent to thin thrombus segment. The tissues were rinsed with cold saline solution and weighed.

### 2.5. Tissue Incubation

In a series of experiments each aneurysm tissue sections were incubated in medium in the presence and absence of DL-homocysteine. Aneurysm fragments were cut into approximately 2 mm pieces. Equal wet weights of the tissue were placed in each well of 12-well plates with serum-free Dulbecco's Modified Eagle's Medium (DMEM) with 4500 mg/L glucose (without phenol red and L-glutamine; Sigma-Aldrich, St. Louis, Missouri, USA). The thick and thin ILTs and adjacent walls were separately incubated without or with 100 *μ*mol/L or 500 *μ*mol/L DL-homocysteine (DL-Homocysteine ≥ 95%, Sigma-Aldrich) at 37°C for 6 h in humidified air with 5% v/v CO_2_. From the wide range of DL-Hcy concentrations mentioned in the literature (10-2000 *μ*mol/L) the two values were selected (as discussed). The incubation times were chosen based on previous studies [29]. Nontreated aneurysm tissues were used in each experiment as controls. After treatment, tissue samples were collected for protein extraction.

### 2.6. Protein Extraction

Incubated AAA wall and ILT samples were pulverized to powder consistency using immersion in liquid nitrogen and a homogenizer. Homogenized tissue was put into tubes containing lysis buffer: 150 mM NaCl; 10 mM Tris(hydroxymethyl)aminomethane hydrochloride (Trizma-HCl) pH 7,4; 1% Triton X-100 Solution and inhibitor (Complete Protease Inhibitor Cocktail Tablets; all chemicals from Sigma-Aldrich). The suspension was incubated on ice for 45 min, followed by centrifugation for 15 min at 14 000 g at 4°C. The supernatant was separated into tubes and frozen at -80°C until use. The protein concentrations in the supernatants was estimated using the Bradford reagent (BioRad, Hercules, California, USA) compared with a bovine-serum-albumin-based standard curve. Samples with concentrations lower than those required in the assay were concentrated by centrifugation (using an Amicon ultra-0.5 centrifugal filter unit; Merck, Kenilworth, New Jersey, United States) and reassayed.

### 2.7. Measurements

#### 2.7.1. Matrix Metalloproteinase Activities

Tissue protein samples were analyzed for MMP-2 and MMP-9 activity (using the SensoLyte® 490 MMP-2 Assay Kit and SensoLyte® Plus 520 MMP-9 Assay Kit; AnaSpec, Fremont, California, USA) according to manufacturer's instructions. Both assays were designed for the detection of MMP activity in a variety of biological samples, e.g., tissue homogenate, and proceeded as follows.

The MMP-2 activity assay used an EDANS/DABCYL fluorescence resonance energy transfer (FRET) peptide. The assay was carried out in black 96-well microplates and assay buffer was used as the background control. Fluorescence intensity was measured (using a Multilabel Plate Reader, PerkinElmer, Waltham, Massachusetts, USA) with excitation and emission wavelengths: 340 nm and 490 nm, respectively. The fluorescence readings (end-point reading of MMP activities) were expressed in relative fluorescence units (RFU) and were converted to *μ*M based on standard curves generated with an EDANS fluorescence reference standard. The *μ*M values were then normalized using the total protein concentration for each sample and results were expressed as *μ*M/mg of total protein.

The MMP-9 activity assay used a fluorometric MMP-9 assay kit with a precoated monoclonal anti-human-MMP-9 antibody. The substrate was a 5-FAM/QXL520 fluorescence resonance energy transfer (FRET) peptide. End-point analysis was performed (using the Multilabel Plate Reader) with excitation at 490 nm and emission at 520 nm. Assay buffer was used as a blank. RFUs were converted to ng based on a standard curve constructed with purified MMP. Results were normalized and expressed as ng/mg of total protein. Assays were carried out in duplicate from the four different tissue segments from each patient.

#### 2.7.2. Haemostatic Factor Concentrations

Enzyme-linked immunosorbent assays (ELISA) were used to measure haemostatic parameters in aortic tissue homogenates. A microplate reader (EnVision Multilabel Plate Reader; PerkinElmer, USA) and manufacturer's instructions of kits (all from AssayPro, St. Charles, Missouri, USA) were followed to determine: Tissue factor activity (AssaySense Human Tissue Factor (TF) Chromogenic Activity Assay Kit); tissue plasminogen activator (t-PA) concentrations (AssayMax Human tPA ELISA kit); plasminogen concentrations (AssayMax Human Plasminogen ELISA kit); plasminogen activator inhibitor 1 (PAI-1) concentrations (AssayMaxTM Human PAI-1 ELISA Kit). The protein concentration in the tissue homogenates was used for value standardisation. The final concentrations of t-PA and PAI-1 were expressed as ng/mg, plasminogen as *μ*g/mg, and TF as pM/mg of total protein extract. Assays were performed in duplicate from the four different tissue pieces from each patient.

### 2.8. Data Analysis

Statistical analysis was performed (using Statistica v.13.0; StatSoft, Tulsa, Oklahama, USA). Continuous variables are presented as means and standard deviations. To compare means between samples incubated with different Hcy concentrations, one-way analysis of variance (ANOVA) was used with least significant difference (LSD) post hoc tests. Correlations between parameters were determined using Pearson correlation coefficients. Statistical significance was set at p < 0.05.

## 3. Results

### 3.1. Total Homocysteine Levels in Cases

Table 1 summarizes the characteristics of patients in the AAA group. HHcy was defined as serum Hcy levels above 15 *μ*mol/L [30]. Thirteen of the 36 (36%) patient with AAA had HHcy.

### 3.2. Effects of DL-Homocysteine Incubation on MMPs and Haemostatic Parameters in Aneurysm Tissues

**Figure 2:**
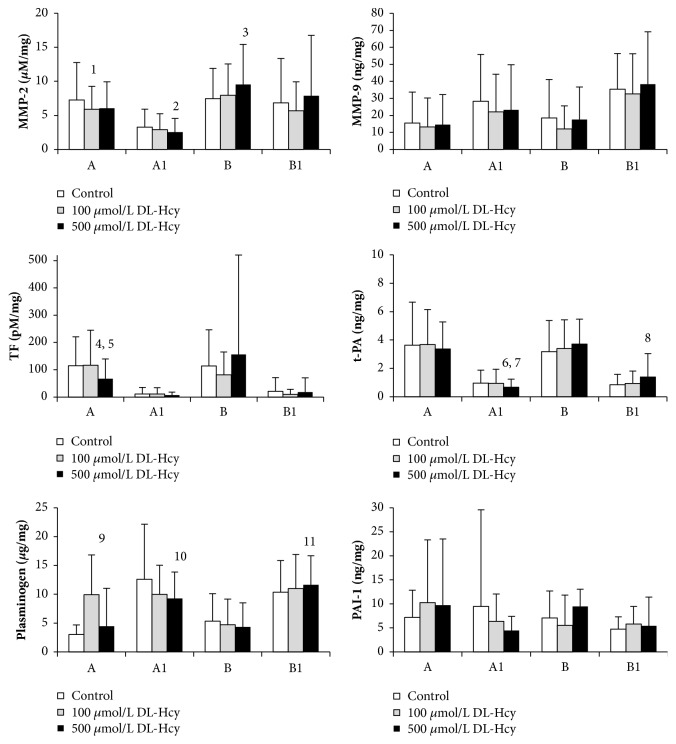
The effect of DL-homocysteine (DL-Hcy) on proteolytic and haemostatic protein expression in thin (B1) and thick (A1) intraluminal thrombus and walls underlying thick (A) and thin (B) intraluminal thrombus (n=36). Sections of aneurysm tissues were incubated in the absence of DL-Hcy (control) and with 100 or 500 *µ*mol/L DL-Hcy for 6 h. Concentrations are per mass of total protein. Bars represent standard deviations. One way ANOVA, with LSD post-hoc tests for pairwise comparisons of control sample and Hcy-treated aneurysm tissues Post-hoc LSD tests: 1, 100 *μ*mol/L DL-Hcy vs control (p=0.046); 2, 500 *μ*mol/L DL-Hcy vs control (p=0.006); 3, 500 *μ*mol/L DL-Hcy vs control (p=0.011); 4, 500 *μ*mol/L DL-Hcy vs control (p=0.007); 5, 500 *μ*mol/L DL-Hcy vs 100 *μ*mol/L DL-Hcy (p=0.005); 6, 500 *μ*mol/L DL-Hcy vs control (p=0.021); 7, 500 *μ*mol/L DL-Hcy vs 100 *μ*mol/L DL-Hcy (p=0.031); 8, 500 *μ*mol/L DL-Hcy vs control (p=0.024); 9, 100 *μ*mol/L DL-Hcy vs control (p=0.004); 10, 500 *μ*mol/L DL-Hcy vs control (p=0.017); 11, 500 *μ*mol/L DL-Hcy vs control (p=0.018).

The results demonstrated that, compared with tissues incubated in medium without DL-Hcy, addition of DL-Hcy had significant effect on MMP-2 and TF activity and plasminogen and t-PA concentrations in a dose-dependent manner with different effects according to different thrombus thicknesses (as described below). Aneurysm tissue exposure to different concentrations of DL-Hcy did not induce any significant changes in MMP-9 activity and PAI-1 concentration in comparison to control aneurysm tissue. Results are displayed in Figure 2.

#### 3.2.1. Thin Aneurysm Sections (B and B1)

Compared with control tissue, DL-Hcy at a concentration of 500 *μ*mol/L was found to significantly increase the active MMP-2 level in wall sections from below thin ILT segments (B) (9.54 ± 5.88 versus 7.44 ± 4.48, p=0.011). When thin thrombus sections (B1) were treated with DL-Hcy, there was an increase in t-PA and plasminogen concentrations compared to control tissue, only at 500 *μ*mol/L (respectively, 1.39 ± 1.65 versus 0.84 ± 0.74, p=0.024; 11.65 ± 5.05 versus 10.34 ± 5.52, p=0.018).

#### 3.2.2. Thick Aneurysm Sections (A and A1)

As shown by fluorescence-quenched cleaving assays, tissue sections from wall adjacent to thick thrombus (A) exposed to 100 *μ*mol/L DL-Hcy showed a significant decrease in active MMP-2 level compared to control tissues (5.89 ± 3.39 versus 7.26 ± 5.49, p=0.046). Thick thrombus section (A1), after treatment with 500 *μ*mol/L DL-Hcy, also showed a decrease in active MMP-2 level (2.53 ± 2.02 versus 3.28 ± 2.65, p=0.006). Sections from walls (A) showed a decrease in TF activity which was statistically significant at 500 *μ*mol/L DL-Hcy, in comparison to control tissues and tissues incubated with 100 *μ*mol/L DL-Hcy (respectively, 67.1 ± 72.6 versus 114.5 ± 106.3, p=0.007; 67.1 ± 72.6 versus 116.8 ± 128.3, p=0.005). t-PA concentrations in thick thrombus sections (A1) were statistical significantly decreased with 500 *μ*mol/L DL-Hcy compared to control tissues (0.67 ± 0.57 versus 0.96 ± 0.91, p=0.021) and compared to 100 *μ*mol/L DL-Hcy (0.67 ± 0.57 versus 0.94 ± 1.00, p=0.031). Plasminogen levels also gradually decreased in thick thrombus segments (A1) after treatment with 500 *μ*mol/L DL-Hcy (9.25 ± 4.59 versus 12.63 ± 9.56, p=0.017). Incubation with 100 *μ*mol/L DL-Hcy induced a significant increase in plasminogen concentrations in sections of walls (A) compared with control samples (6.93 ± 6.88 versus 3.01 ± 1.66, p=0.004).

### 3.3. The Association of Serum Hcy with AAA Diameter, ILT Thickness, and Hemostatic Parameters

A significant positive Pearson correlation was found between the aneurysm diameter and thrombus thickness (r=0.688, p=0.0001). Significant correlations were found between serum Hcy and TF in thin thrombus (B1; r=0.653, p=0.0001) and t-PA in wall adjacent to thick ILT (A; r=0.583, p=0.0001) (both tissues incubated in medium without Hcy) (Table 2). No other significant correlations were observed between serum Hcy and measured parameters.

### 3.4. Correlations between Hemostatic and Proteolytic Parameters within ILT and AAA Wall Sections

Correlations between pairs of parameters measured after incubation in the presence or absence of Hcy with samples from sites A, B, A1, and B1 are listed in Table 3 and the significant values (p<0.05) are highlighted. Analysis revealed positive correlations at all sites of the AAA between the activities/concentrations of MMP-2, TF, and PAI-1, measured in control samples and after incubation with 100 and 500 *μ*mol/L DL-Hcy. Interestingly, within the wall section adjacent to thick ILT (A), no significant correlations were found between plasminogen concentrations measured in control tissue and after incubation with different concentrations of DL-Hcy. Furthermore, no correlations were observed for MMP-9 activity at B1 sites (thin ILT).

## 4. Discussion

Previously published studies have demonstrated that there is evidence for an association between homocysteine levels and abdominal aortic aneurysms [7, 8]. It is suggested that extracellular matrix degradation due to altered activities of metalloproteinases and the haemostatic system that may be induced by Hcy is one of the key steps in AAA formation, further dilatation, and eventually rupture [6].

The main finding of this study is that incubation of aneurysm tissue with Hcy could influence the activity or concentration of MMPs and haemostatic parameters in a concentration-dependent manner which differed according to thrombus thickness. Moreover, we observed that activities/levels of most haemostatic and proteolytic parameters in control samples (tissues incubated in absence of Hcy) were strongly correlated with activities/levels after incubation with different concentrations of Hcy. The same pattern was observed for most measured parameters (except for plasminogen concentrations at A sites (wall underlying thick ILT) and MMP-9 activity at B1 sites (thin ILT)). This may indicate that the effects of Hcy depend to a large extent on initial activities/concentrations of these factors in both ILTs and adjacent walls, and the high activity of proteolytic and hemostatic factors in the plasma of patients in combination with elevated Hcy levels could potentially lead to intensification of wall degradation processes and consequently increase the risk of AAA rupture.

In the presented work, it was observed that Hcy increased MMP-2 activities and also plasminogen and t-PA concentrations in thin segments of AAAs (B and B1) in a dose-dependent manner. Maximal activities/concentrations were reached at 500 *μ*mol/L of Hcy. In contrast, it was found that incubation of thick AAA sections (A and A1) with Hcy decreased MMP-2 and TF activities and plasminogen and t-PA concentrations in a dose-dependent manner. These results may reflect locational differences in the way in which Hcy influences haemostatic and proteolytic processes. This implies that Hcy may be implicated in AAA pathophysiology and activates or stimulates the deposition of haemostatic and proteolytic parameters mostly in the blood-rich luminal layer of an ILT, at the bloodstream interface which is biologically active. In our previous study we demonstrated that intensified proteolytic degradation of aortic walls and a higher activity of coagulation occurred in the thin segments of AAAs [28]. Presumably, as these processes may be induced by factors originating from plasma including Hcy, it seems that the thin areas of the AAA are the most exposed to Hcy's influence. Furthermore, thin thrombus allows more effective penetration of plasma factors and inflammatory cells from the lumen towards the aneurysm wall, than thick segments [25]. It should be noted that ILTs have different types and these are structurally different from type to type and may progress from one to another [31]. An ILT has a blood-rich luminal layer, at the bloodstream interface, which is biologically active as a result of neutrophil recruitment, accumulation of platelets, formation of fibrin, and retention of plasminogen and t-PA. Over time an ILT luminal layer progresses into an intermediate layer and eventually to a dead abluminal region at the AAA wall, following erythrocyte lysis, leukocyte apoptosis, and various degrees of fibrinolysis. In the oldest abluminal layers enzymatic activities are usually very low [32]. It has been reported that only the luminal layer of an ILT contains active proteins and has higher procoagulant activity, and that levels of active proteases decrease through the depth of the ILT [28, 32]. Moreover, absence of cellular content in the deeper layers of the ILT (thick ILT) makes synthesis of new proteases in these areas impossible. In addition, it is worth noticing that ILT thickness cannot be assumed to be uniform throughout the entire aneurysm [20, 21]. ILT may vary in thickness within each individual aneurysm. Thus, it is likely that in the samples analyzed in the presented work thin sections contained predominantly luminal (bioactive) regions, whereas thicker multilayered sections may have had a combination of distinct layers with low but measurable amounts of active proteases in the luminal layer. It is possible that enhanced proteolysis and impaired fibrinolysis caused by Hcy in thin AAA segments may be a part of a chain reaction leading to the destruction of the AAA wall and finally resulting in its rupture, a hypothesis which needs further research.

It is known that aortic wall damage, in association with an AAA, leads to the exposure of TF, which in turn activates coagulation processes and ILT formation. Degradation of the aortic wall may also play a role in the activation of fibrinolysis by t-PA. As a result of these processes, plasmin is generated, an activator of MMPs, important in the sequence of events leading to the destruction of the aortic matrix in an AAA. Some studies suggest that increased Hcy in blood may lead to alterations in these processes, and most of these observations suggest that HHcy may lead to impaired thrombosis, fibrinolysis, and proteolysis [15–18, 33, 34].

Paradoxically, our study found that Hcy affected active MMP-2 levels while no effect on MMP-9 activity was reported. The lack of any association and demonstrated effect of Hcy on MMP-9 activity may argue against a role for Hcy in MMP-9 activation. Nevertheless, Hcy-induced activation of latent MMP-9 has been previously shown. Lee et al. [35] reported that Hcy enhances MMP-9 production in murine macrophages. Moshal et al. [14] observed that Hcy induces MMP-9 in microvascular endothelial cells (MVECs). Moreover, Tsarouhas et al. [36] reported a positive association between both tissue and serum MMP-9 concentrations and serum Hcy levels and suggested a role of Hcy in AAA pathogenesis by regulating the expression of MMP-9. An explanation for this discrepancy may be found in part by the dual effect exerted by Hcy. Bescond et al. [13] reported that high experimental concentrations of Hcy inhibit the activation of MMPs at a high molar ratio. On the other hand lower Hcy levels increase MMP-2 and MMP-9 expression levels and activate proMMP-2 at a low molar ratio. It should be noted that despite the selection of the Hcy concentration range based on data from available literature, the concentrations used in the presented study significantly exceed those occurring in the plasma of the healthy population. The Hcy concentrations (100 and 500 *μ*mol/L) used in the present study would correspond to conditions of intermediate or severe HHcy [37].

Our results revealed no correlation between the size of the AAA and levels of total serum Hcy. This observation confirms the results of Lindqvist et al. [38] who did not find any association between AAA and homocysteine, but contrasts with the results of other investigations [8, 10, 39]. Most previous measurements of circulating levels of Hcy have found higher levels in the AAA patients compared to levels in control groups without AAA [9, 10, 39]. It should be also mentioned that in other case-control studies the mean plasma Hcy was higher in AAA patients than in our report [5, 8–10]. This discrepancy may stem from the various sample sizes of the previous reports. In our work only two significant correlations with serum Hcy were observed: with TF activities in control samples of thin ILTs (B1) and with t-PA concentration within control wall adjacent to thick ILT (A). The first association may be related to the characteristics of thin thrombus, which conveys more stimulating factors involved in proteolysis from blood to the AAA wall, while thick sections of ILTs constitute a barrier for those factors. However, Wiernicki et al. [40] observed an association of thinner ILT with lower plasma Hcy levels and this may be related to the potential role of Hcy as a prothrombotic marker. Regarding the correlation between serum Hcy and t-PA in wall section underlying thick ILT, previous observations have shown preferential localization of t-PA exactly within the aneurysm wall [4, 22]. Furthermore, in our previous work we found that t-PA was detectable in the highest concentrations in the AAA wall, especially in walls adjacent to thick ILT [28]. It is worth noting that our study measurements were conducted with ILT and AAA wall tissue fragments without conditioned media. Factors may be less or more retained within tissues due to differential binding to various structural components or released to the media. Unfortunately, released proteins were not considered in this study.

It is suggested that the relationship between the occurrence of an AAA and an elevated Hcy concentration is complex, because this may be influenced by environmental and genetic factors, which may also explain discrepancies between obtained results [6]. It is not known whether Hcy is involved in the initiation of AAA pathogenesis, or whether it intensifies changes in vessel walls caused by other factors. It is suggested that this amino acid may be an additional factor whose action is superimposed on the harmful effects of others. If Hcy's participation in the pathogenesis of AAA is confirmed, it would be possible to introduce appropriate conservative treatment in patients at risk. Whether Hcy plays a role in aneurysm formation and/or in aneurysm expansion or whether Hcy is simply a marker of the condition needs to be investigated.

There are several limitations to this study. The presented experiment was carried out in vitro, and a number of limitations associated with this type of experiment do not allow a simple translation of the results obtained into the processes occurring in the body. An in vivo environment would be much more complex, with a significantly higher extent of proteolytic and haemostatic activity. It should be also noted that the relative proportions of the biologically active regions of the biopsies may differ between samples, and assessment of full thickness homogenates normalized to total protein, may artefactually influence the overall expression and activity measurements of luminal components. Moreover, consideration of AAA wall section without ILT would benefit the study and strengthen the results.

## 5. Conclusions

In spite of the limitations mentioned, we have demonstrated that incubation of aneurysm tissues with varying concentrations of Hcy showed a significant influence on fibrinolytic and proteolytic parameters, depending on thrombus thickness, in all examined AAA sites. This in vitro study showed a possible link between increased Hcy levels and activation of MMP-2 and haemostatic factors in thin sections of an aneurysm. Considering the significant ability of fibrinolytic and coagulative systems to regulate extracellular matrix proteolysis of the AAA wall, knowledge concerning the differential effects of Hcy depending on the spatial distribution of components of the two systems may help in the planning of surveillance of AAAs, to evaluate the potential risk of rupture and possibly contribute to a better strategy in therapy. However, the significance of this observation is uncertain as some tissue fragments were exposed to extreme levels of Hcy, although this does indicate the potential for excess Hcy to enhance damage of arterial elastin in thinner AAA segments as a result of increased activity of MMPs and other fibrinolytic parameters. Further investigations are required to clarify the specific mechanisms of the relationship between the effects of Hcy and the formation of AAAs.

## Figures and Tables

**Table 1:** Demographic, clinical, and biochemical characteristics in subjects with an abdominal aortic aneurysm (AAA).

	AAA (n=36)
Age (years)	71 ± 8
Gender (male/female)	27 (75%)/9 (25%)
Current smoker	17 (47%)
Diabetes mellitus	7 (19%)
Hypertension	23 (64%)
CVD, Myocardial infarction	2 (6%)
CVD, Stroke	2 (6%)
DVT	1 (3%)

Total Hcy (*μ*mol/L)	13.96 ± 6.70

AAA diameter (mm)	59 ± 13
ILT thickness (mm)	32 ± 10

Values are means ± standard deviations (continuous variables) or counts with percentages (categorical variables); CVD, cardiovascular disease; DVT, deep vein thrombosis; Hcy, homocysteine; ILT, intraluminal thrombus.

**Table 2:** Correlations between serum homocysteine (Hcy) level and abdominal aortic aneurysm (AAA) size, intraluminal thrombus thickness (ILT), and other parameters in control samples.

Parameter	Pearson's correlation coefficient	p
Hcy vs maximum AAA size	-0.952	0.575
Hcy vs ILT thickness	-0.117	0.492
Maximum AAA diameter vs ILT thickness	0.688	$\underline{0.0001}$
Hcy vs TF (B1)	0.653	$\underline{0.0001}$
Hcy vs t-PA (A)	0.583	$\underline{0.0001}$

TF, tissue factor; t-PA, tissue plasminogen activator. Measurement units are given in Figure 1. Underlined values show significant correlations. A = wall adjacent to thick thrombus segment; B1 = thin thrombus section.

**Table 3:** Correlations between parameters measured within tissue sections incubated in the absence of DL-homocysteine (DL-Hcy) (control) and the same parameters measured within thin and thick intraluminal thrombus (ILT) and adjacent walls of abdominal aortic aneurysm (AAA) sections after incubation with different concentrations of DL-Hcy.

		**AAA wall parts adjacent to ILT**				**Intraluminal thrombus**			
		**Site A **		**Site B**		**Site A1**		**Site B1**	
**Parameter**	**Correlation**	**r**	**p**	**r**	**p**	**r**	**p**	**r**	**p**

**MMP-2**	**control vs 100 ** ***μ*** **mol/L Hcy**	$\underline{0.697}$	$\underline{0.0001}$	$\underline{0.358}$	$\underline{0.030}$	$\underline{0.843}$	$\underline{0.0001}$	$\underline{0.742}$	$\underline{0.0001}$
	**control vs 500 ** ***μ*** **mol/L Hcy**	$\underline{0.468}$	$\underline{0.003}$	$\underline{0.493}$	$\underline{0.002}$	$\underline{0.662}$	$\underline{0.0001}$	$\underline{0.745}$	$\underline{0.0001}$
**MMP-9**	**control vs 100 ** ***μ*** **mol/L Hcy**	$\underline{0.432}$	$\underline{0.008}$	0.208	0.217	$\underline{0.568}$	$\underline{0.0001}$	−0.062	0.717
	**control vs 500 ** ***μ*** **mol/L Hcy**	$\underline{0.689}$	$\underline{0.0001}$	$\underline{0.587}$	$\underline{0.0001}$	0.238	0.156	−0.041	0.650
**TF**	**control vs 100 ** ***μ*** **mol/L Hcy**	$\underline{0.574}$	$\underline{0.0001}$	$\underline{0.594}$	$\underline{0.0001}$	$\underline{0.904}$	$\underline{0.0001}$	$\underline{0.740}$	$\underline{0.0001}$
	**control vs 500 ** ***μ*** **mol/L Hcy**	$\underline{0.634}$	$\underline{0.0001}$	$\underline{0.402}$	$\underline{0.014}$	$\underline{0.749}$	$\underline{0.0001}$	$\underline{0.926}$	$\underline{0.0001}$
**t-PA**	**control vs 100 ** ***μ*** **mol/L Hcy**	$\underline{0.783}$	$\underline{0.0001}$	$\underline{0.559}$	$\underline{0.0001}$	$\underline{0.763}$	$\underline{0.0001}$	$\underline{0.396}$	$\underline{0.015}$
	**control vs 500 ** ***μ*** **mol/L Hcy**	0.303	0.073	$\underline{0.519}$	$\underline{0.001}$	$\underline{0.501}$	$\underline{0.002}$	0.168	0.321
**Plasminogen**	**control vs 100 ** ***μ*** **mol/L Hcy**	0.114	0.503	$\underline{0.508}$	$\underline{0.001}$	0.311	0.065	$\underline{0.815}$	$\underline{0.0001}$
	**control vs 500 ** ***μ*** **mol/L Hcy**	−0.082	0.628	$\underline{0.731}$	$\underline{0.0001}$	$\underline{0.347}$	$\underline{0.036}$	$\underline{0.564}$	$\underline{0.0001}$
**PAI-1**	**control vs 100 ** ***μ*** **mol/L Hcy**	$\underline{0.525}$	$\underline{0.001}$	$\underline{0.582}$	$\underline{0.0001}$	$\underline{0.379}$	$\underline{0.021}$	$\underline{0.734}$	$\underline{0.0001}$
	**control vs 500 ** ***μ*** **mol/L Hcy**	$\underline{0.389}$	$\underline{0.017}$	$\underline{0.446}$	$\underline{0.006}$	$\underline{0.369}$	$\underline{0.025}$	$\underline{0.779}$	$\underline{0.0001}$

r, p, Pearson's correlation coefficient and p-value; MMP-2,9, matrix metalloproteinase 2 or 9; TF, tissue factor; t-PA, tissue plasminogen activator; and PAI-1, plasminogen activator inhibitor-1. Site A = wall adjacent to thick ILT; site B = wall adjacent to thin ILT; site A1 = thick ILT; site B1 = thin ILT. Measurement units are given in Figure 1. Underlined values show significant correlations.

## Data Availability

The data used to support the findings of this study are available from the corresponding author upon request.

## References

[1] Alexander J. J. (2004). The pathobiology of aortic aneurysms. *Journal of Surgical Research*.

[2] Hobeika M. J., Thompson R. W., Muhs B. E., Brooks P. C., Gagne P. J. (2007). Matrix metalloproteinases in peripheral vascular disease. *Journal of Vascular Surgery*.

[3] Schneiderman J., Bordin G. M., Engelberg I. (1995). Expression of fibrinolytic genes in atherosclerotic abdominal aortic aneurysm wall. A possible mechanism for aneurysm expansion. *The Journal of Clinical Investigation*.

[4] Houard X., Rouzet F., Touat Z. (2007). Topology of the fibrinolytic system within the mural thrombus of human abdominal aortic aneurysms. *The Journal of Pathology*.

[5] Sofi F., Marcucci R., Giusti B. (2005). High levels of homocysteine, lipoprotein (a) and plasminogen activator inhibitor-1 are present in patients with abdominal aortic aneurysm. *Thrombosis and Haemostasis*.

[6] Moroz P., Le M. T. Q., Norman P. E. (2007). Homocysteine and abdominal aortic aneurysms. *ANZ Journal of Surgery*.

[7] Halazun K., Bofkin K., Asthana S., Evans C., Henderson M., Spark J. (2007). Hyperhomocysteinaemia is associated with the rate of abdominal aortic aneurysm expansion. *Journal of Vascular Surgery*.

[8] Wong Y. Y. E., Golledge J., Flicker L. (2013). Plasma total homocysteine is associated with abdominal aortic aneurysm and aortic diameter in older men. *Journal of Vascular Surgery*.

[9] Cao H., Hu X., Zhang Q. (2014). Homocysteine level and risk of abdominal aortic aneurysm: a meta-analysis. *PLoS ONE*.

[10] Warsi A. A., Davies B., Morris-Stiff G., Hullin D., Lewis M. H. (2004). Abdominal aortic aneurysm and its correlation to plasma homocysteine, and vitamins. *European Journal of Vascular and Endovascular Surgery*.

[11] Hämelahti P., Järvinen O., Sisto T. (2002). Methylenetetrahydrofolate reductase gene C677T mutation is related to the defects in the internal elastic lamina of the artery wall. *European Journal of Clinical Investigation*.

[12] Moat S. J., Lang D., McDowell I. F. W. (2004). Folate, homocysteine, endothelial function and cardiovascular disease. *The Journal of Nutritional Biochemistry*.

[13] Bescond A., Augier T., Chareyre C., Garçon D., Hornebeck W., Charpiot P. (1999). Influence of homocysteine on matrix metalloproteinase-2: Activation and activity. *Biochemical and Biophysical Research Communications*.

[14] Moshal K. S., Singh M., Sen U. (2006). Homocysteine-mediated activation and mitochondrial translocation of calpain regulates MMP-9 in MVEC. *American Journal of Physiology-Heart and Circulatory Physiology*.

[15] Fryer R. H., Wilson B. D., Gubler D. B., Fitzgerald L. A., Rodgers G. M. (1993). Homocysteine, a risk factor for premature vascular disease and thrombosis, induces tissue factor activity in endothelial cells. *Arteriosclerosis, Thrombosis, and Vascular Biology*.

[16] Hajjar K. A., Mauri L., Jacovina A. T. (1998). Tissue plasminogen activator binding to the annexin II tail domain: Direct modulation by homocysteine. *The Journal of Biological Chemistry*.

[17] Khajuria A., Houston D. S. (2000). Induction of monocyte tissue factor expression by homocysteine: A possible mechanism for thrombosis. *Blood*.

[18] Midorikawa S., Sanada H., Hashimoto S., Watanabe T. (2000). Enhancement by homocysteine of plasminogen activator inhibitor-1 gene expression and secretion from vascular endothelial and smooth muscle cells. *Biochemical and Biophysical Research Communications*.

[19] Kazi M., Thyberg J., Religa P. (2003). Influence of intraluminal thrombus on structural and cellular composition of abdominal aortic aneurysm wall. *Journal of Vascular Surgery*.

[20] Di Martino E. S., Bohra A., Geest J. P. V., Gupta N., Makaroun M. S., Vorp D. A. (2006). Biomechanical properties of ruptured versus electively repaired abdominal aortic aneurysm wall tissue. *Journal of Vascular Surgery*.

[21] Speelman L., Schurink G. W. H., Bosboom E. M. H. (2010). The mechanical role of thrombus on the growth rate of an abdominal aortic aneurysm. *Journal of Vascular Surgery*.

[22] Fontaine V., Jacob M.-P., Houard X. (2002). Involvement of the mural thrombus as a site of protease release and activation in human aortic aneurysms. *The American Journal of Pathology*.

[23] Koole D., Zandvoort H. J. A., Schoneveld A. (2013). Intraluminal abdominal aortic aneurysm thrombus is associated with disruption of wall integrity. *Journal of Vascular Surgery*.

[24] Vorp D. A., Lee P. C., Wang D. H. J. (2001). Association of intraluminal thrombus in abdominal aortic aneurysm with local hypoxia and wall weakening. *Journal of Vascular Surgery*.

[25] Adolph R., Vorp D. A., Steed D. L., Webster M. W., Kameneva M. V., Watkins S. C. (1997). Cellular content and permeability of intraluminal thrombus in abdominal aortic aneurysm. *Journal of Vascular Surgery*.

[26] Wiernicki I., Stachowska E., Safranow K. (2010). Enhanced matrix-degrading proteolytic activity within the thin thrombus-covered wall of human abdominal aortic aneurysms. *Atherosclerosis*.

[27] Siennicka A., Zuchowski M., Kaczmarczyk M., Cnotliwy M., Clark J. S., Jastrzebska M. (2016). Spatial differences of matrix metalloproteinase-2 and matrix metalloproteinase-9 within abdominal aortic aneurysm wall and intraluminal thrombus. *Journal of Physiology and Pharmacology*.

[28] Siennicka A., Zuchowski M., Kaczmarczyk M., Cnotliwy M., Clark J. S., Jastrzębska M. (2018). Tissue factor levels and the fibrinolytic system in thin and thick intraluminal thrombus and underlying walls of abdominal aortic aneurysms. *Journal of Vascular Surgery*.

[29] Doronzo G., Russo I., Del Mese P. (2010). Role of NMDA receptor in homocysteine-induced activation of Mitogen-Activated Protein Kinase and Phosphatidyl Inositol 3-Kinase pathways in cultured human vascular smooth muscle cells. *Thrombosis Research*.

[30] Christopher R., Nagaraja D., Shankar S. K. (2007). Homocysteine and cerebral stroke in developing countries. *Current Medicinal Chemistry*.

[31] O'Leary S. A., Kavanagh E. G., Grace P. A., McGloughlin T. M., Doyle B. J. (2014). The biaxial mechanical behaviour of abdominal aortic aneurysm intraluminal thrombus: classification of morphology and the determination of layer and region specific properties. *Journal of Biomechanics*.

[32] Folkesson M., Silveira A., Eriksson P., Swedenborg J. (2011). Protease activity in the multi-layered intra-luminal thrombus of abdominal aortic aneurysms. *Atherosclerosis*.

[33] Da Cunha A. A., Scherer E., Da Cunha M. J. (2012). Acute hyperhomocysteinemia alters the coagulation system and oxidative status in the blood of rats. *Molecular and Cellular Biochemistry*.

[34] Ke X. D., Foucault-Bertaud A., Genovesio C., Dignat-George F., Lamy E., Charpiot P. (2010). Homocysteine modulates the proteolytic potential of human arterial smooth muscle cells through a reactive oxygen species dependant mechanism. *Molecular and Cellular Biochemistry*.

[35] Lee S. J., Lee Y. S., Seo K. W. (2012). Homocysteine enhances MMP-9 production in murine macrophages via ERK and Akt signaling pathways. *Toxicology and Applied Pharmacology*.

[36] Tsarouhas K., Tsitsimpikou C., Apostolakis S. (2011). Homocysteine and metalloprotease-3 and - 9 in patients with ascending Aorta Aneurysms. *Thrombosis Research*.

[37] Krishna S. M., Dear A., Craig J. M., Norman P. E., Golledge J. (2013). The potential role of homocysteine mediated DNA methylation and associated epigenetic changes in abdominal aortic aneurysm formation. *Atherosclerosis*.

[38] Hosoda T. (2012). C-kit-positive cardiac stem cells and myocardial regeneration. *American Journal of Cardiovascular Disease*.

[39] Brunelli T., Prisco D., Fedi S. (2000). High prevalence of mild hyperhomocysteinemia in patients with abdominal aortic aneurysm. *Journal of Vascular Surgery*.

[40] Wiernicki I., Millo B., Safranow K., Gorecka-Szyld B., Gutowski P. (2011). MMP-9, Homocysteine and CRP Circulating Levels Are Associated with Intraluminal Thrombus Thickness of Abdominal Aortic Aneurysms–New Implication of the Old Biomarkers. *Disease Markers*.

